# Interval cancers in a national colorectal screening programme based on faecal immunochemical testing: Implications for faecal haemoglobin concentration threshold and sex inequality

**DOI:** 10.1177/09691413231188252

**Published:** 2023-07-19

**Authors:** Gavin RC Clark, Thomas Godfrey, Calum Purdie, Judith Strachan, Francis A Carey, Callum G Fraser, Robert JC Steele

**Affiliations:** 1 Centre for Research into Cancer Prevention and Screening, University of Dundee, 59805 Ninewells Hospital and Medical School, Dundee, UK; 2 9571 Public Health Scotland, Edinburgh, UK; 3 Blood Sciences and Scottish Bowel Screening Laboratory, 59805 Ninewells Hospital and Medical School, Dundee, UK; 4 Department of Pathology, 59805 Ninewells Hospital and Medical School, Dundee, UK

**Keywords:** colonoscopy, colorectal cancer screening, faecal immunochemical test, faecal haemoglobin, faecal occult blood test, interval cancers

## Abstract

**Objective:**

To compare interval cancer proportions (ICP) in the faecal immunochemical test (FIT)-based Scottish Bowel Screening Programme (SBoSP) with the former guaiac faecal occult blood test (gFOBT)-based SBoSP and investigate associations between interval cancer (IC) and faecal haemoglobin concentration (f-Hb) threshold, sex, age, deprivation, site, and stage.

**Methods:**

The ICP data from first year of the FIT-based SBoSP and the penultimate year of the gFOBT-based SBoSP were compared in a prospective cohort design.

**Results:**

With FIT, 801 colorectal cancers (CRCs) were screen detected (SDC), 802 were in non-participants, 548 were ICs, 39 were colonoscopy missed and 72 were diagnosed after incomplete screening; with gFOBT: 540, 904, 556, 45, and 13, respectively. FIT had a significantly higher proportion of SDC compared to IC than gFOBT. For FIT and gFOBT, ICP was significantly higher in women than men. As f-Hb threshold increased, ICP increased and, for any f-Hb threshold for men, a lower threshold was required for comparable ICP in women. In Scotland, the current threshold of ≥80 µg Hb/g faeces would have to be lowered to ≥40 µg Hb/g faeces for women to achieve sex equality for ICP. In the FIT-based SBoSP, there were four times as many stage I SDC than IC. This was reversed in advanced stages, with twice as many stage IV CRC diagnosed as IC versus SDC.

**Conclusions:**

Reducing the numbers of IC requires lowering the f-Hb threshold. Using different f-Hb thresholds for women and men could eliminate the sex disparity, but with additional colonoscopy.

## Introduction

In a colorectal cancer (CRC) screening programme based on regular testing, the term “interval cancer“ (IC) is defined, after a review by the World Endoscopy Organization, as ‘colorectal cancer diagnosed after a colorectal screening examination or test in which no cancer is detected, and before the date of the next recommended exam’.^1^ By this definition, in a CRC screening programme that employs biennial testing for haemoglobin in faeces, an IC is a CRC that has been diagnosed at any time in the 2-year period following a negative test result. It does not refer to a cancer that presents after a normal colonoscopy carried out due to a positive test result. Traditionally, this is referred to as a missed cancer;^2^ however, a recent review has proposed that IC should include those that might arise due to: a missed lesion (prior examination adequate or prior examination negative but inadequate), a detected lesion not resected or incompletely resected, or a failed biopsy identification.^3^

Whilst it may be possible for a CRC to develop *de novo* within a 2-year period, particularly in the younger population screened,^4^ this is likely to be very rare, and most ICs will be cancers that have not been detected by the screening test, either at an earlier or pre-malignant (adenomatous) stage. Consequently, the proportion of cancers diagnosed in the population undergoing screening that are ICs is an important indicator of the quality of a population screening programme, since it provides a surrogate measure of test sensitivity, that is, the proportion of cancers in the population that leads to a positive test result.

In Scotland, a national CRC screening programme, based on an initial guaiac faecal occult blood tests (gFOBT) (*hema-screen*, Immunostics, Inc., Ocean, NJ, USA), began with three pilot screening rounds between 1 March 2000 and 31 May 2007,^2^ after which it was rolled out across the whole country for the 50 to 74 age range.^5^ From 1 July 2010 to 12 January 2011, a pilot evaluation of a quantitative faecal immunochemical test (FIT) was carried out in 2 of the 14 NHS Boards responsible for regional healthcare. During this pilot, instead of the usual gFOBT/qualitative FIT algorithm, in which *hema-screen* SPECIFIC (Immunostics, Inc.) was used as a follow-up test for those with a weak-positive gFOBT result,^6,7^ invitees were sent a quantitative FIT (OC Sensor, Eiken Chemical Co., Ltd, Tokyo, Japan) as the initial investigation and the faecal haemoglobin concentration (f-Hb) threshold chosen to trigger an invitation to colonoscopy was ≥80 μg Hb/g faeces.^8^ As a result of this FIT pilot, the Scottish Government decided to replace the initial gFOBT with a single FIT as the primary screening test for the Scottish Bowel Screening Programme (SBoSP) and, after a tendering process, the HM-JACKarc (Minaris Medical Co, Ltd, Tokyo, Japan) was chosen, with the ≥80 μg Hb/g faeces threshold retained. The FIT-based SBoSP started on 20 November 2017, and results from the first year of this programme, along with a comparison with the previous gFOBT-based programme, have been published.^9^

In this article, the characteristics of the ICs that arose in participants in the first year of the FIT-based SBoSP are described and compared with the ICs that arose during the previous initial gFOBT-based SBoSP.

## Methods

The methods employed for the gFOBT pilot, the gFOBT-based SBoSP, the quantitative FIT pilot, and the quantitative FIT programme in Scotland have been described in detail previously.^2,5–9^ Briefly, in the current SBoSP, biennial screening using a single quantitative FIT (HM-JACKarc, Minaris Medical Co., Ltd, Tokyo, Japan) at an f-Hb threshold of ≥80 μg Hb/g faeces, and offered to all 50-year-olds to 74-year-olds, commenced on 20 November 2017. Individuals aged over 74 years can opt in to the SBoSP by request, though data from these individuals were not included in this study. FIT specimen collection devices were sent out in the mail from the Scottish Bowel Screening Centre, along with pictorial information, and returned by the participant for analysis in the Scottish Bowel Screening Laboratory which has International Organization for Standardization (ISO) 15,189 accreditation. Individuals with a test result of ≥80 μg Hb/g faeces (a ‘positive’ test result) were invited for further investigation, usually colonoscopy, by their respective NHS Board.

Any CRC diagnosed at any time between a ‘negative’ test result (i.e. f-Hb <80 μg Hb/g faeces) and the next invitation to participate *or* within the 2 years after a negative test result (whichever came first) was classified as an IC. To identify the IC, all records on the screening database were linked with cases of CRC confirmed in the Scottish Cancer Registry (SCR), and the time between the date of generation of the test result and the date of diagnosis of the CRC was calculated. The SCR employs multiple sources, and case ascertainment is nearly complete.^10^

Other definitions used are as follows. Screen-detected cancer (SDC): a CRC diagnosed as a result of a colonoscopy following a positive screening test result. Non-participant cancer (NPC): a CRC diagnosed in an individual who had not completed a test in the round in question. Colonoscopy missed cancer (CMC): a CRC diagnosed within 2 years of a negative colonoscopy carried out consequent on a positive screening test result. Cancer where screening was incomplete (CSI): a CRC diagnosed in an individual with a positive test result but where there was no indication of follow up.

IC incidence rate was calculated as the number of interval cancers per 100,000 person-years at risk. The term ‘person-years at risk’ was derived by taking the number of negative screening test results in the cohort, multiplied by the number of years of follow up, that is, two.

When examining IC proportion (ICP) by f-Hb threshold, the total number of cancers detected in the screening population was held constant, and the ratio of IC to SDC varied assuming all CRC below a given f-Hb threshold would be detected as IC, and all CRC at or above that f-Hb threshold would be SDC.

The IC data derived from the SBoSP between 20 November 2017 and 31 October 2018 were compared with those from 20 November 2015 to 31 October 2016 of the gFOBT programme so that, taking account of the biennial invitation cycle, the populations being studied were as comparable as possible.

A cancer located between (and including) the caecum and the splenic flexure (ICD-10 codes C180–C185) was defined as a right-sided colonic cancer, whilst a cancer located between (and including) the descending colon and sigmoid colon (ICD-10 codes C186–C187) was defined as a left-sided colonic cancer. A cancer in the rectum or at the recto-sigmoid junction (ICD-10 codes C19–C20) was defined as rectal.

CRC staging was based on the TNM system^11^ as follows: stage I: T1-2, N0, M0; stage II: T3-4, N0, M0; stage III: T1-4, N1-2, M0; and stage IV: T1-4, N0-2, M1. Polyp cancers that had been completely removed at colonoscopy with no further surgery were classified as stage I.

Socio-economic deprivation status was derived from the Scottish Index of Multiple Deprivation (SIMD)^12^ which is based on small geographical data zones that are identified by postcode. Version 2016 was used for invitations prior to 2017 and version 2020 for invitations from 2017 onwards. SIMD allows relative ranking and is usually expressed as quintiles, with quintile 5 being the least deprived and quintile 1 the most deprived.

The statistical significance of the distribution of results was tested using the chi-squared test.

## Results

**Figure 1.**
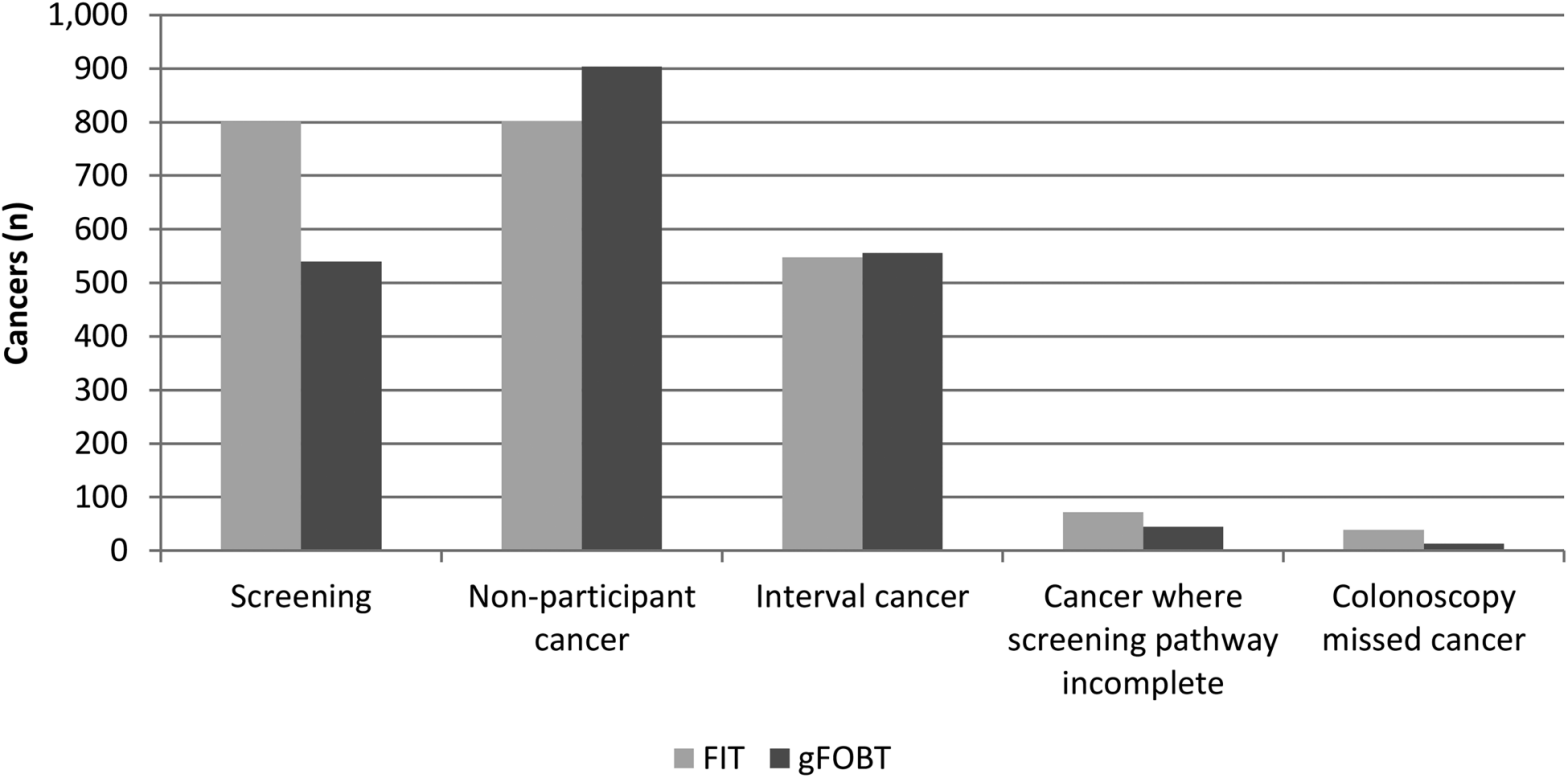
Routes of detection of colorectal cancers in the population screened, for faecal immunochemical tests (FIT) and guaiac faecal occult blood tests (gFOBT).

In the population offered screening with quantitative FIT in the study period, 801 CRCs were diagnosed through screening (SDC), 802 arose in NPC, 548 were IC, 39 were CMC, and 72 were cancers diagnosed after an incomplete screening pathway (CSI), as shown in Figure 1. For the gFOBT period, SDC, NPC, IC, CMC, and CSI were 540, 904, 556, 45, and 13, respectively (Figure 1). Thus, the FIT-based screening SBoSP was associated with a significantly higher proportion of SDC and significantly lower proportion of IC (40.6%, 95% confidence interval (CI): 38.0%–43.3%) than for gFOBT (50.7%, 95% CI: 47.8%–53.7%). There were 569,382 negative tests for FIT and 476,132 for gFOBT, giving an IC incidence rate per 100,000 persons screened of 48.1and 58.4, respectively.

**Figure 2.**
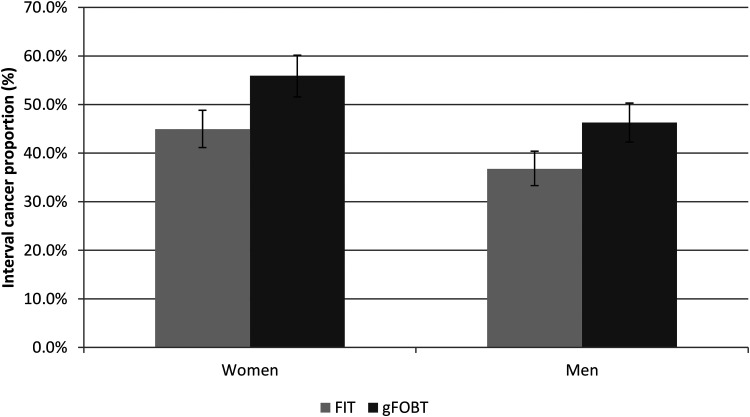
Interval cancer proportions (%) and 95% confidence intervals, for faecal immunochemical tests (FIT) and guaiac faecal occult blood tests (gFOBT), by sex.

For both FIT (p < 0.001) and gFOBT (p < 0.001), the ICP was significantly higher in women than in men, although the differences were very similar for FIT and gFOBT (Figure 2). The ICP increased with increasing age up to those aged 65 to 69 years with gFOBT, but a similar pattern was not seen with FIT (Supplemental Figure 1) and there was no discernible variation in IC proportion with socio-economic deprivation with either FIT or gFOBT (Supplemental Figure 2).

Table 1 shows the proportions of SDC and IC at the different stages for both FIT and gFOBT-based screening. In the FIT-based programme, the proportion of stage I CRCs that were SDC (78.4%) was about four-fold that observed in IC (21.6%). This distribution was reversed in the more advanced stages, with about twice as many stage IV CRCs being IC (68.2%). In the gFOBT-based SBoSP, the pattern was similar, but the proportion of stage I CRCs that were SDC (68.5%) was only twice that in the IC group. Table 1 also shows the proportions of SDC and IC at the different anatomical sites for both FIT and gFOBT-based programmes, and the patterns were very similar.

**Table 1.** Screen-detected cancer (SDC, %) and interval cancer (IC, %) proportions, for faecal immunochemical tests (FIT) and guaiac faecal occult blood tests (gFOBT), by stage and site.

		FIT SDC		FIT IC		gFOBT SDC		gFOBT IC	
		n	%	n	%	n	%	n	%
Stage	I	287	78.4	79	21.6	220	68.5	101	31.5
	II	188	58.6	133	41.4	137	50.6	134	49.4
	III	190	57.8	139	42.2	140	48.8	147	51.2
	IV	64	31.8	137	68.2	38	20.9	144	79.1
	Unknown	72	54.5	60	45.5	5	14.3	30	85.7
Site	Right sided	264	49.2	273	50.8	174	38.4	279	61.6
	Left sided	244	72.0	95	28.0	167	62.8	99	37.2
	Rectum	280	62.6	167	37.4	195	54.6	162	45.4
	Unknown	13	50.0	13	50.0	4	20.0	16	80.0
Total		801	100	548	100	540	100	556	100

The proportion numerator is the number of IC and the denominator is the number of SDC plus IC.

**Figure 3.**
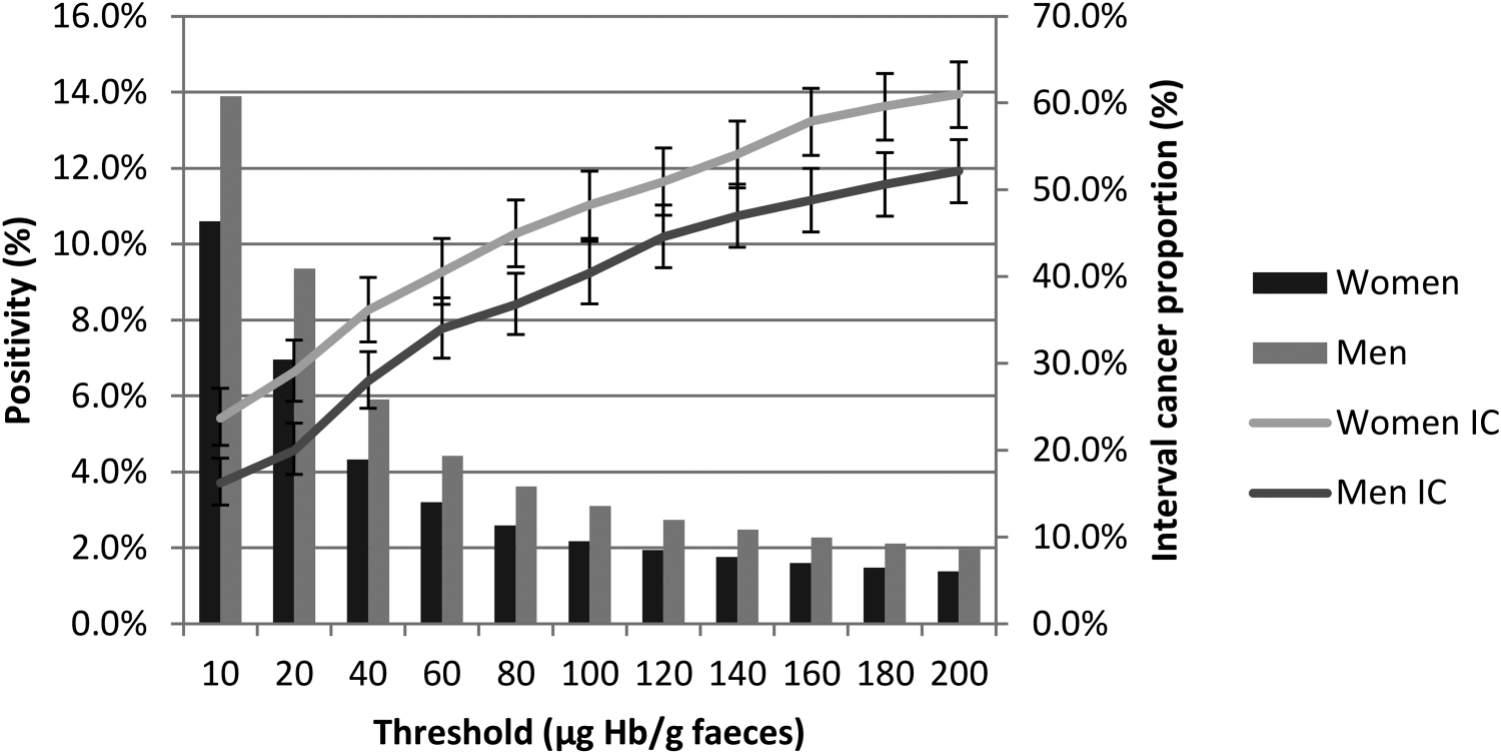
Positivity (%) and interval cancer (IC, with 95% confidence intervals) proportion (%) for women and men, by varying faecal haemoglobin concentration (µg Hb/g faeces) thresholds for positivity.

In the FIT-based screening population, as the f-Hb (i.e. the threshold for further investigation) increased, the ICP increased, and this increase was roughly parallel in women and men (Table 2 and Figure 3). By visual inspection, a lower threshold is required to achieve the same ICP in women as for any given f-Hb threshold for men. For example, in the SBoSP, which uses a threshold of ≥80 μg Hb/g faeces, to obtain sex equality in terms of IC a threshold of around 40 μg Hb/g faeces would have to be introduced for women.

**Table 2.** Test positivity (%) and interval cancer (IC) proportion (%) for all, men, and women by faecal haemoglobin concentration threshold for positivity.

	Positivity (%)			IC proportion (%)		
Threshold (≥ μg Hb/g faeces)	All	Men	Women	All	Men	Women
**10**	12.2	13.9	10.6	19.7	16.2	23.7
**20**	8.1	9.4	7.0	24.2	20.0	29.0
**40**	5.1	5.9	4.3	31.8	28.0	36.1
**60**	3.8	4.4	3.2	37.1	34.0	40.5
**80**	3.1	3.6	2.6	40.6	36.8	45.0
**100**	2.6	3.1	2.2	44.1	40.4	48.3
**120**	2.3	2.7	1.9	47.6	44.6	50.9
**140**	2.1	2.5	1.8	50.3	47.0	54.1
**160**	1.9	2.3	1.6	53.1	48.8	57.9
**180**	1.8	2.1	1.5	54.9	50.6	59.6
**200**	1.7	2.0	1.4	56.3	52.2	61.0

The overall test positivity obtained with FIT at a threshold of ≥80 μg Hb/g faeces was 3.1% whereas that with gFOBT was 2.2%. Thus, as can be seen from Table 2, an equivalent positivity would have been obtained by FIT at an f-Hb threshold of around 140 μg Hb/g faeces, which would have resulted in an ICP for FIT of 50.3%, very similar to the gFOBT ICP of 50.7%. For women, the 140 μg Hb/g faeces threshold would have given 1.8% positivity with an ICP of 54.1% vs. 55.9% for gFOBT; for men, the 140 μg Hb/g faeces threshold would have given 2.5% positivity with an ICP of 47.0% vs 46.3% for gFOBT.

## Discussion

We have previously reported on the transition from gFOBT to FIT at an f-Hb threshold of ≥80 μg Hb/g faeces in the SBoSP,^9^ and demonstrated an increased screen-detection rate for both CRC and adenomas, but at the cost of a lower positive predictive value for CRC and increased colonoscopy demand caused by a combination of increased uptake and higher test positivity. In a recent Scottish report on pathways to diagnosis of CRC, the high false-negative rate of gFOBT was discussed, and analysis of the equivalent with FIT suggested.^13^ We are now able to report on aspects of the IC for the first year of FIT-based screening since sufficient time has elapsed to allow accumulation of reliable data in the SCR. As well as the identification of interval cancers, the addition of SCR linkage, in combination with a longer period of follow up, led to better ascertainment of the number of SDC than reported previously, with a greater number identified in the time period.^9^

All aspects of gFOBT and FIT were recently compared in a Cochrane Review^14^ and in a meta-analysis (in which data from 29 studies were used to compare the IC in FIT-based or gFOBT-based CRC screening).^15^ A lower IC incidence was found after a negative FIT result compared to a negative gFOBT, and for every IC from FIT there were 2.6 SDC compared to 1.2 for gFOBT. This translates to an ICP in the screened population of 27.8% for FIT and 45.5% for gFOBT. However, these results were generated by pooling heterogenous data, and the f-Hb threshold varied from ≥10 to ≥200 μg Hb/g faeces with a median of ≥20 μg Hb/g faeces, which, globally, is the most frequently used f-Hb threshold, as reported in a recent review.^16^ A study in France confirmed these findings and revealed a dramatic decrease in the cumulative incidence rates of IC after transition to FIT from gFOBT.^17^

However, several countries, including the UK and The Netherlands, where again it has been shown that the proportion of IC in FIT-based screening appears to be lower than that with gFOBT,^18^ have CRC population screening programmes using FIT at f-Hb thresholds higher than ≥20 μg Hb/g faeces to cope with colonoscopy capacity. Indeed, within the UK, where health is devolved to the four nations, three different thresholds are employed for this reason: ≥ 80 μg Hb/g faeces in Scotland, ≥ 120 μg Hb/g faeces in England, and ≥150 μg Hb/g faeces in Wales and Northern Ireland. Therefore, because the clinical sensitivity of the test is dependent on the f-Hb threshold applied, it is important to assess IC in the context of any individual programme since these provide key information with which to monitor performance. This concept is supported by the results of a study from The Netherlands in which the cumulative incidence of CRC after a negative result from an FIT and the sensitivity of the FIT for detection of CRC at low (≥15 μg Hb/g faeces) and high (≥47 μg Hb/g faeces) f-Hb thresholds were compared.^19^

In Scotland, where an f-Hb threshold of ≥80 μg Hb/g faeces is currently adopted, the ICP was 40.6% (95% CI: 38.0%–43.3%) for FIT compared with 50.7% (95% CI: 47.8%–53.7%) for gFOBT, so that, although not performing as well as it might at a lower f-Hb threshold, FIT has proven itself to be superior to gFOBT by this metric. Interestingly, it has been demonstrated that there is a positive correlation between f-Hb and risk of IC even at very low f-Hb.^20^

In terms of inequalities, we found no associations between FIT-based screening ICP and either age or socio-economic variation (as determined using the SIMD), but, as we have already observed in gFOBT screening, FIT at a single f-Hb threshold for all participants results in higher ICP in women than in men.^21^ Why this should be is not entirely clear, but it is known that in a screened population women have lower f-Hb than men,^22^ the possible reasons for which have been discussed elsewhere,^23^ and it may simply be that, for women, proportionally more bleeding is required from a CRC to pass a given f-Hb threshold.

In any event, the data presented here make it clear that for FIT to have the same clinical sensitivity as estimated by ICP in women and men, sex-partitioned thresholds should be employed. In the first year of the SBoSP, using a threshold of about 40 μg Hb/g faeces for women and retaining the current threshold at ≥80 μg Hb/g faeces for men would have resulted in an almost identical ICP for both sexes assuming equal uptake of colonoscopy after a positive test result. Interestingly, equalising the positivity, a solution which we proposed in a previous communication,^23^ would only have required a reduction in the threshold for women to ≤50 μg Hb/g faeces, but this would not have equalised the ICP. Lowering the threshold to ≤40 μg Hb/g faeces would have resulted in an increase in positivity in women from 2.6% to 4.3% which would have required approximately 4050 further screening colonoscopies throughout the year across the country, representing a 67.0% increase over the 6048 carried out on women in that period. An alternative strategy would be to calculate the f-Hb thresholds for men and women that would keep the colonoscopy requirement constant and the ICP the same, but this would inevitably result in a raising of the threshold and thus inferior outcomes for men.

It is interesting to note that in the Swedish CRC screening programme in Stockholm/Gotland between October 2015 and September 2017, where the invited age range was 60 to 69 years and differential thresholds of ≥40 and ≥80 μg Hb/g faeces were used for women and men, respectively, the ICP was 25.2% for women and 38.0% for men^24^; since the test sensitivity was higher and the IC rate lower in women, it was suggested that it might be appropriate to lower the f-Hb threshold in men. Similarly, in Denmark, in a study aimed at finding age-specific and sex-specific f-Hb thresholds that could improve population-based CRC screening, it was concluded that, in an FIT-based CRC screening programme, it is possible to decrease the number of colonoscopies required while at the same time increasing overall sensitivity and specificity, and detect more cancers and adenomas by using different f-Hb thresholds for different female and male age groups. This does, however, increase inequality in sensitivity.^25^ Thus, it is clear that adjusting thresholds for women and men in different geographical locations can have different effects. This may be related to the well-established variation in population f-Hb concentrations in different countries.^26^ Moreover, the results of the study reported here were generated using the HM-JACKarc FIT system, which is used in Scotland, Wales and Northern Ireland, and in Iberia and Asia, whereas most other data on IC have been generated using the OC-Sensor, and it is well documented that different FIT systems give different numerical results.^27^ This is the first comprehensive report on IC using the HM-JACKarc.

The use of IC data to approximate sensitivity has several limitations as described elsewhere.^19^ One is that CRC missed at the point of screening may not present symptomatically, participants may also die of another cause prior to the end of the interval; both of these would over-estimate sensitivity. Conversely, an IC following screening may have been at a pre-cancerous stage at the point of screening, under-estimating sensitivity for CRC.

From a UK perspective, our data are of importance as they provide an indication of what ICPs are likely to be experienced with the different thresholds used across the four countries. In Wales and Northern Ireland with an f-Hb threshold of ≥150 μg Hb/g faeces the ICP might be expected to be around 51%. However, as the Swedish experience illustrates, the same threshold may give different results in different regions, compounded by the fact, as documented above, that different FIT analytical systems do give different results^27^ with even pre-analytical factors such as ambient temperature at time of specimen collection affecting FIT results.^28^ Further, in the same population, the ICP differed between gFOBT, a pilot evaluation of FIT, and an FIT-based programme, again demonstrating that data from one FIT system may not be transferable to another,^29^ although global efforts to standardise results from different FIT analytical systems are underway.^30^ It should therefore be standard practice for a CRC screening programme to monitor its own ICP, both to assess the quality of the programme and to allow transparent information to be communicated to participants who have received a negative test result.

The significance of a substantial ICP is highlighted by the data on stage at diagnosis which show that ICs are substantially less likely to be stage I and more likely to be at more advanced stages. This is very similar to our previous findings in the gFOBT-based SBoSP,^2^ and underlines the importance of working to reduce the numbers of ICs, primarily by decreasing the f-Hb threshold. Also, in keeping with our findings with gFOBT, ICs in the FIT-based SBoSP were more likely to be situated in the right colon and rectum than in the left colon.

Finally, despite the clear advantages of FIT over gFOBT in terms of specificity for human haemoglobin and analytical performance characteristics, when FIT was used at the f-Hb threshold that gave the same positivity as gFOBT, the ICP was essentially the same.^29^ This means that the main advantage of FIT over gFOBT is its quantitative power, and it must be appreciated that increased sensitivity for CRC is dependent on lowering the f-Hb threshold and that this in turn impacts on colonoscopy requirement. It may be that strategies to stratify screening participants using variables such as age, gender, socio-economic status, screening history, and genetic susceptibility will increase the diagnostic accuracy of FIT-based screening, or that totally new screening modalities will replace FIT, but at present the only sure way of reducing the number of ICs is to reduce the f-Hb threshold. Stratification using different f-Hb thresholds for women and men could certainly eliminate the current sex disparity but, unless men are to be disadvantaged in the context of current programmes, this can only be achieved at the cost of more colonoscopies. On the basis of a commissioned economic analysis, the UK National Screening Committee has reported that the most cost-effective f-Hb threshold is likely to be ≥20 μg Hb/g faeces,^31^ which, in the SBoSP, would give a positivity of 8.1%: it is to be hoped that, in the UK, our National Health Services can rise to this challenge.

## Contributors

GRCC collected and analysed the data, performed the statistical tests, and contributed to writing the paper. TG assisted with collection and analysis of the data and contributed to writing the paper. CP assisted with collection and analysis of the data and contributed to writing the paper. JS supervised the laboratories that analysed the gFOBT and FIT in the SBoSP and contributed to writing the paper. FAC provided the necessary pathology data and contributed to writing the paper. CGF contributed to interpretation of the data and significantly to the writing of the paper. RJCS is clinical director of the SBoSP, conceived and supervised the study, contributed to interpretation of the data, and wrote first draft of the paper. All authors have seen and approved the final submission.

## Supplemental Material

sj-docx-1-msc-10.1177_09691413231188252 - Supplemental material for Interval cancers in a national colorectal screening programme based on faecal immunochemical testing: Implications for faecal haemoglobin concentration threshold and sex inequality Supplemental material, sj-docx-1-msc-10.1177_09691413231188252 for Interval cancers in a national colorectal screening programme based on faecal immunochemical testing: Implications for faecal haemoglobin concentration threshold and sex inequality by Gavin RC Clark, Thomas Godfrey, Calum Purdie, Judith Strachan, Francis A Carey, Callum G Fraser and Robert JC Steele in Journal of Medical Screening

sj-docx-2-msc-10.1177_09691413231188252 - Supplemental material for Interval cancers in a national colorectal screening programme based on faecal immunochemical testing: Implications for faecal haemoglobin concentration threshold and sex inequality Supplemental material, sj-docx-2-msc-10.1177_09691413231188252 for Interval cancers in a national colorectal screening programme based on faecal immunochemical testing: Implications for faecal haemoglobin concentration threshold and sex inequality by Gavin RC Clark, Thomas Godfrey, Calum Purdie, Judith Strachan, Francis A Carey, Callum G Fraser and Robert JC Steele in Journal of Medical Screening
