# Supplementary material for: Interval cancers in a national colorectal screening programme based on faecal immunochemical testing: Implications for faecal haemoglobin concentration threshold and sex inequality
Source: J Med Screen. 2023 Jul 19;31(1):21–7. doi: 10.1177/09691413231188252 (PMC10878005; doi:10.1177/09691413231188252)
Supplement: sj-docx-1-msc-10.1177_09691413231188252 - Supplemental material for Interval cancers in a national colorectal screening programme based on faecal immunochemical testing: Implications for faecal haemoglobin concentration threshold and sex inequality [file sj-docx-1-msc-10.1177_09691413231188252.docx]

Supplementary Figure 1 – interval cancer proportions (%), with 95% confidence intervals, for faecal immunochemical tests (FIT) and guaiac faecal occult blood tests (gFOBT), by five-year age band.
